# Normative kinematics of reaching and dexterity tasks: moving towards a quantitative baseline for Functional Capacity Evaluations (FCEs)

**DOI:** 10.1080/23335432.2017.1326843

**Published:** 2017-12-15

**Authors:** Angelica E. Lang, Clark R. Dickerson

**Affiliations:** a Department of Health Science, University of Saskatchewan, Saskatoon, Canada; b Faculty of Applied Health Sciences, Department of Kinesiology, University of Waterloo, Waterloo, Canada

**Keywords:** Functional Capacity Evaluation, normative data, kinematics, upper limb

## Abstract

**Purpose:** This work generates a comprehensive description of upper extremity and torso kinematics of a healthy population during reaching and dexterity Functional Capacity Evaluation (FCE) tasks. **Methods:** Upper limb and torso kinematic data were collected from 30 young, healthy participants as they performed three common FCE tasks: repetitive reaching, fingertip dexterity, and hand and forearm dexterity. Kinematic profiles were created for all clinically relevant angles of the torso, shoulder, elbow, and wrist. **Results:** These provocative tasks require large ranges of motion and create high demand postures for the upper limb, specifically at the shoulder. Arm elevation was up to 90°, while humeral internal rotation of 25° was observed. Torso angles were typically below 30° from neutral and elbow flexion remained within 90°–120° for nearly all tasks. Wrist ulnar deviation ranged from 0° to 26° for both wrists. **Conclusion:** The normative data created in this investigation provide a description of healthy motion during reaching and dexterity tasks. These normative curves are the initial step towards understanding movement that would contraindicate return to work during an FCE. This work supports a future clinical goal of being able to identify persons at risk of further injury or disability if returned to work too early*.*

## 1. Introduction

Functional Capacity Evaluations (FCE) are objective, standardized batteries of physical performance and functional measures that are used to establish the ability to perform work-related tasks (King et al. 1998; Gross & Battié 2003). The specific purposes for FCE’s are to improve the likelihood that an injured worker will be safe in future work performance, to identify functional limitations so they can be resolved or worked around by return to work modifications, and to determine the presence and level of disability to aid in legal or insurance cases (Matheson 1996). Function-centered rehabilitation as guided by FCE’s can decrease time to return-to-work and the likelihood of lost time sequelae after time loss injuries (Oesch et al. 2006; Kool et al. 2007).

One popular FCE tool is the WorkWell System (WWS) (formally the Isernhagen Work System). There are several types of tasks involved in the WWS that can be used to evaluate a wide range of work-related injuries, including a lifting evaluation with specific parameters, dexterity tasks or ambulation tasks (Isernhagen 1992), and normative capacity values exist for comparison of capacity outcomes (Soer et al. 2009). Further, this system has comparatively low equipment requirements, allowing for evaluations that are more directly relatable to the workplace. Finally, the WWS has been demonstrated to have good reliability and construct validity (Hart 1988; Reneman et al. 2002; Reneman, Fokkens, et al. 2005; Brouwer et al. 2003; Gross & Battié 2003), with one review reporting the WWS to have the highest reliability and predictive validity among different commercially available FCE systems (Gouttebarge et al. 2004).

An essential aspect of the FCE process is the evaluator’s ability to interpret the worker’s performance. Interpretation of capacity outcomes is guided by normative capacity data or results from a job demands analysis (Soer et al. 2009), but the interpretation of body mechanics and posture for many tasks lacks clear guidance. Possessing normative typical upper extremity kinematic data from FCE tasks enhances understanding of movement during these tasks, making the kinematic components of FCE’s more straightforward to interpret and improves the consistency of the return to work evaluation process. These data provide a baseline for comparison for future analyses of injured populations, such as those with rotator cuff tear repairs or breast cancer survivors (Lin et al. 2011; McClure et al. 2006), helping evaluators to more readily and reliably recognize pathological or atypical motion. The identification of atypical motion can then be used to guide treatment or to determine potential job modifications to decrease risk of recurrent injury and return the worker to the job sooner.

Several aspects of performance are considered by evaluators during FCEs. Body mechanics, compensatory movements, changes in speed or control of movement, muscle tremor, facial expressions, and competitive test behaviors are prime examples (Chappell et al. 2006). Thus, evaluators need to determine which specific performance attributes merit closest monitoring. Normative kinematic data can help evaluators direct their attention to those aspects of motion that typify healthy mechanics, making FCEs and return to work decisions more reliable.

Some previous guidelines for observation of typical mechanics during FCEs exist, but they are nearly exclusively for floor to waist lifting tasks (Smith 1994; Reneman, Fokkens, et al. 2005). In addition, aspects of the established definitions are vague. For instance, for non-lifting tasks, observation criteria directs evaluators to classify functional abilities of a patient based on their deviation from normal (Trippolini et al. 2014) but almost no description of normal movement is provided for comparison. Normative capacity data created from healthy populations is the gold standard for comparison of capacity outcomes of FCEs (Bhambhani et al. 1994; Soer et al. 2009) and kinematic alterations caused by injury are often determined by comparing to normal, healthy controls (Winter 1991; McClure et al. 2006; Roy et al. 2008; Lomond & Côté 2011), but the same information for comparison of movement strategies during FCE task performance is not available. Normal movement, used as a best measure to identify alterations in kinematics that could be caused by injury, must first be clearly documented and understood before deviations caused by injury or work intensity can be identified.

The purpose of this project was to quantify upper extremity kinematics of a control population during select reaching and dexterity tasks of a FCE, thus providing a comprehensive description of normative upper extremity movement strategies during said tasks and also characterizing FCE task upper extremity movements at a higher resolution than previously accomplished.

## 2. Methods

### 2.1. Participants

Thirty participants (15 males; mean (standard deviation) age = 23 (1.76) years, height = 1.70 (0.10) m, body mass = 72.9 (13.65) kg, QuickDASH = 4.17 (3.95)) participated. Participants were excluded if they reported any upper extremity or back pain during functional tasks or any injuries to the upper extremities or back in the last six months. The study protocol was approved by the University of Waterloo Research Ethics Board and all participants provided written informed consent.

### 2.2. Experimental protocol

Participants performed three reaching and dexterity tasks that targeted upper extremity motions based on the WWS FCE protocol (Reneman, Soer, et al. 2005; Gross 2006). The tasks were always performed in the same order; Repetitive Reaching (RR) task, Fingertip Dexterity (FD) task, and then Hand and Forearm Dexterity (HFD) task (Table 1).

**Table 1.** Order of task and subtask performance.

Task	Subtask
Repetitive reaching	Right to Left, Right Hand
	Right to Left, Left Hand
	Left to Right, Right Hand
	Left to Right, Left Hand
Fingertip dexterity	Right Hand
	Left Hand
	Both Hands
	Assembly
Hand and forearm dexterity	Placing
	Turning

#### 2.2.1. Repetitive reaching task (RRT)

To setup the RR task, two bowls (14 cm diameter) were positioned at the wingspan extrema of each participant on a table adjusted to just below participant elbow height based on the NIOSH light manual materials handling guidelines (Cohen et al. 1997). While sitting, the participant moved the marbles horizontally from one bowl to the other in both directions and with each arm, for a total of four different subtasks:

(1) Right Hand, Left to Right
(2) Left Hand, Left to Right
(3) Right Hand, Right to Left
(4) Left Hand, Right to Left

Each subtask was repeated three times. The participant was instructed to move the marbles as quickly as possible. The measurement of performance was the average time of all three sets of each subtask.

#### 2.2.2. FD task

The Purdue Peg Board Test was used for the FD task (Lafayette Instrument 2002). It includes a peg board with two vertical rows of holes, and pins, washers, and collars that are located along the top. The test apparatus was positioned on a table adjusted to just below participant elbow height when sitting (Cohen et al. 1997). The participant sat in front of the peg board and placed the pins as quickly as possible into the holes in four different subtasks:

(1) Right Hand
(2) Left Hand
(3) Both Hands
(4) Assembly

Each subtask was repeated three times. The final performance measure was the average score of all three sets of each subtask.

#### 2.2.3. HFD task

The Minnesota Manual Dexterity Test was used for the HFD task (Lafayette Instrument 1999). It includes 60 blocks and a folding board with 60 round holes. The HFD is comprised of two test batteries (Surrey et al. 2003):

(1) Placing
(2) Turning

The equipment was positioned on table adjusted to just below elbow height while the participants were sitting. Each participant was instructed to move the blocks as quickly as possible and the total time to complete all subtasks of each task was recorded. Each subtask was repeated three times. The final performance measure was the total time of all three sets of each subtask.

### 2.3. Motion capture

All movements were tracked using 8 VICON MX20 (Vicon Motion Systems, Oxford, UK) optoelectronic infrared cameras positioned around the collection space. Twenty-two individual passive reflective markers were placed on the skin near bony anatomical landmarks on the arms, torso, and pelvis (Table 2). Additionally, five rigid clusters (totaling 17 markers) were placed on the upper extremities and pelvis. Marker three-dimensional positions were sampled at 50 Hz.

**Table 2.** Anatomical landmark locations of individual markers.

Marker	Description
SS	Suprasternal notch
C7	Spinous process of the 7th cervical vertebra
XP	Xiphoid process
T8	Spinous process of the 8th thoracic vertebra
AR*	Acromion
ME*	Medial epicondyle of the humerus
LE*	Lateral epicondyle of the humerus
RS*	Radial styloid
US*	Ulnar styloid
MC2*	2nd metacarpal phalangeal joint
MC5*	5th metacarpal phalangeal joint
IC*	Iliac crest
GT*	Greater trochanter of the femur

* Indicates bilateral placement.

### 2.4. Data analysis

Movement cycles were defined within each trial to facilitate result amalgamation and communication. For all subtasks, a movement cycle was defined as the time during which the arm moved from the starting position and back. The starting position for all tasks, except the HFD turning task, was when the hand was picking up the marble, pin, or block to begin moving it to the next bowl or next spot in the board. The HFD turning task was indivisible, and thus was analyzed holistically.

**Figure 1.**
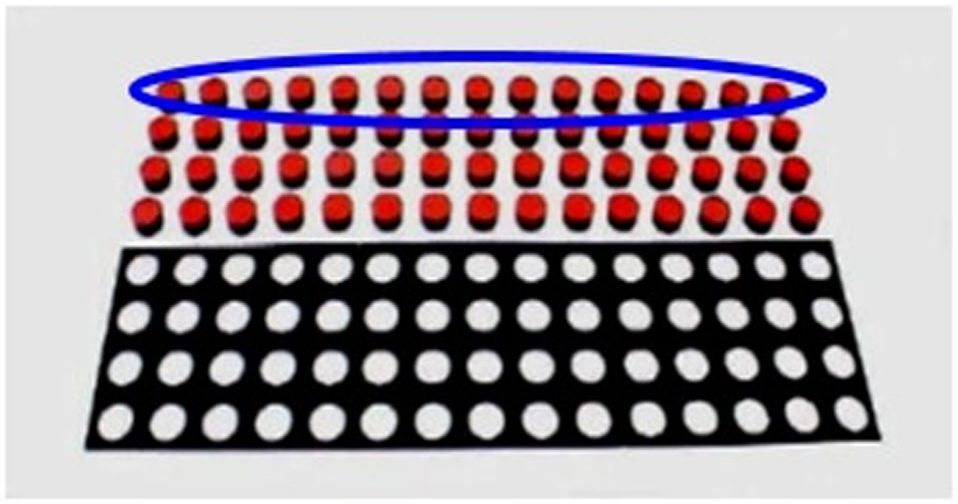
HFD placing task setup.

Note: For this subtask, only the cycles involving movement of the blocks in the row circled were used for analysis.

The start and end points of each cycle were identified through the use of equipment reference markers. An equipment calibration was performed prior to task performance during which reflective markers were placed at a criterion position relative to the task equipment for each task. Cycles were identified by locating when the hand markers passed the value of the position of the equipment calibration marker in the direction of movement. For example, for the FD task, the equipment calibration was performed by placing markers at the edge of the pin storage area and then the *X* (forward/backward direction) value of the marker was extracted. Each time the hand marker passed the *X* value during a trial, the frame number was determined and used to create cycles. All cycles were normalized to the same length and ensemble averaged within each set, with the exception of the HFD placing task. In the placing task, there were four levels of positions for blocks (Figure 1). Only cycles during which the blocks from the highest level were moved were averaged. This task was also divided into thirds and cycles within each third were ensemble averaged. For the RR, FD, and HFD turning task, all sets within a subtask were averaged.

Motion capture data were used to calculate time-varying Euler angles of the torso, humerus, elbow and wrist. The rotation sequence for Euler decompositions for the torso, elbow, and wrist was *Z*-*X*′-*Y*′′ based on the International Society Biomechanics recommendations (Wu et al. 2005). The humerus rotation sequence was chosen to be *X*-*Z*′-*Y*′′ to reflect more clinically relevant angles and address singularity issues (Phadke et al. 2011). Time series joint angle profiles were generated by ensemble averaging all participant curves, and mean, maximum, and minimum values were calculated. The means with +/− one standard deviation for each task or subtask were plotted to create graphical references for the computed profiles (Winter 2009).

One-way ANOVAs were used to test sex effects on capacity scores and each dependent variable.

## 3. Results

The mean capacity scores, in seconds or number of pins, had no sex differences (*p* = 0.1014–0.9649) and equaled or exceeded reported norms (Soer et al. 2009) (Table 3).

**Table 3.** Mean capacity scores for males and females during each FCE subtask.

Task (performance measure)	Males [mean (SD)]	Females [mean (SD)]
Repetitive reaching, Right to Left, Right Hand (s)	56.31 (7.47)	54.61 (9.76)
Repetitive reaching, Right to Left, Left Hand (s)	57.31 (9.65)	54.47 (10.76)
Repetitive reaching, Left to Right, Right Hand (s)	56.18 (9.01)	52.62 (10.14)
Repetitive reaching, Left to Right, Left Hand (s)	57.94 (9.07)	54.29 (11.27)
Fingertip dexterity, Right Hand (# of pins)	17.22 (1.62)	18.16 (1.86)
Fingertip dexterity, Left Hand (# of pins)	16.29 (1.54)	17.1 (2.32)
Fingertip dexterity, Both Hands (# of pins)	13.53 (1.33)	14.28 (1.65)
Fingertip dexterity, assembly (# of pins)	35.77 (6.47)	39.2 (4.39)
Hand and forearm dexterity, placing (s)	197.13 (21.23)	192.47(20.65)
Hand and forearm dexterity, turning (s)	148.67 (17.46)	148.37 (19.46)

### 3.1. Sex

Kinematic results are presented together for males and females, as only a small percentage of tests resulted in significant outcomes, indicating a high probability of findings due to Type 1 errors.

### 3.2. Kinematics

#### 3.2.1. Repetitive reaching task

**Figure 2.**
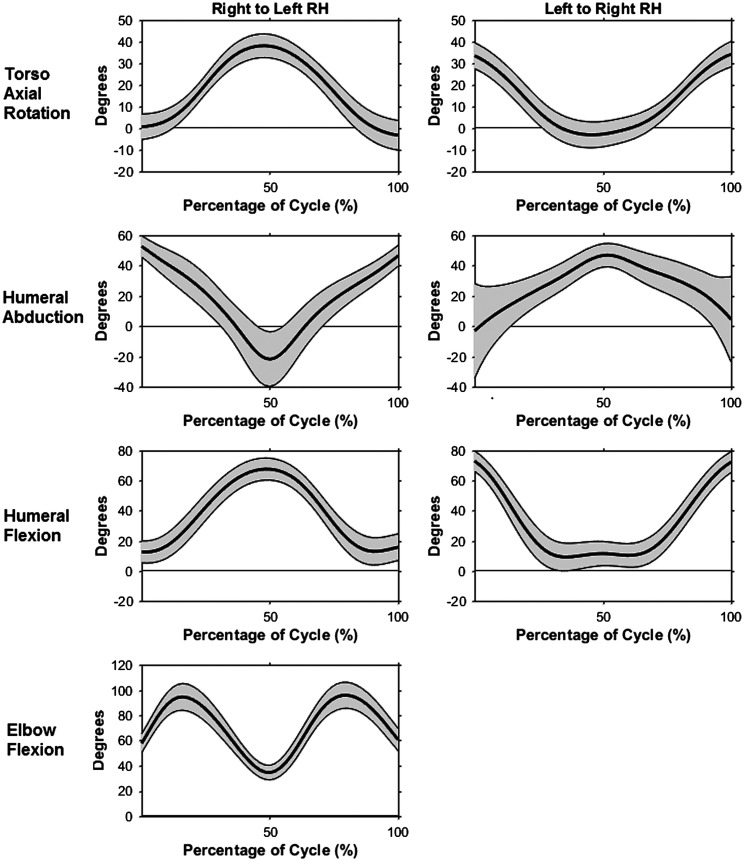
Kinematic profiles with mean (bold line) +/− one standard deviation (gray shaded area) during the right to left, right hand and left to right, right-hand RR subtasks: torso + left/− right axial rotation (top), right humeral abduction (second), right humeral flexion (third), and right elbow flexion (bottom).

Notes: In the same direction/hand sub tasks, humeral abduction peaks during marble pick up and humeral flexion peaks during marble drop off but during the opposite direction/hand the pattern is reverse. The pattern of motion for the elbow was the same for all subtasks.

The RR task required relatively large ranges of motion for both the torso and upper arms. Mean torso flexion remained relatively constant at an average of 16.1° throughout each cycle and subtasks. Participants used a high average absolute range of motion of 40.1° of torso axial rotation for each subtask, although the relative angle range of axial rotation varied with movement direction and hand (Figure 2). Average thoracohumeral abduction peaked at 51.2°, while average flexion maximum was 70.6° (Figure 2). Due to the setup of the task, when abduction was at a maximum, flexion was a minimum, with the reverse also being true. Regardless of hand used or direction of movement, elbow angle was consistent during the RR task (Figure 2), with peaks of 96.4° for the right and 105.9° for the left arm, respectively. Wrist motion had no consistent pattern of movement during any RR subtasks. Flexion/extension angle remained neutral, while ulnar deviation was an average of 7.46° and 24.92° for the right and left wrists, respectively.

#### 3.2.2. Fingertip dexterity

**Figure 3.**
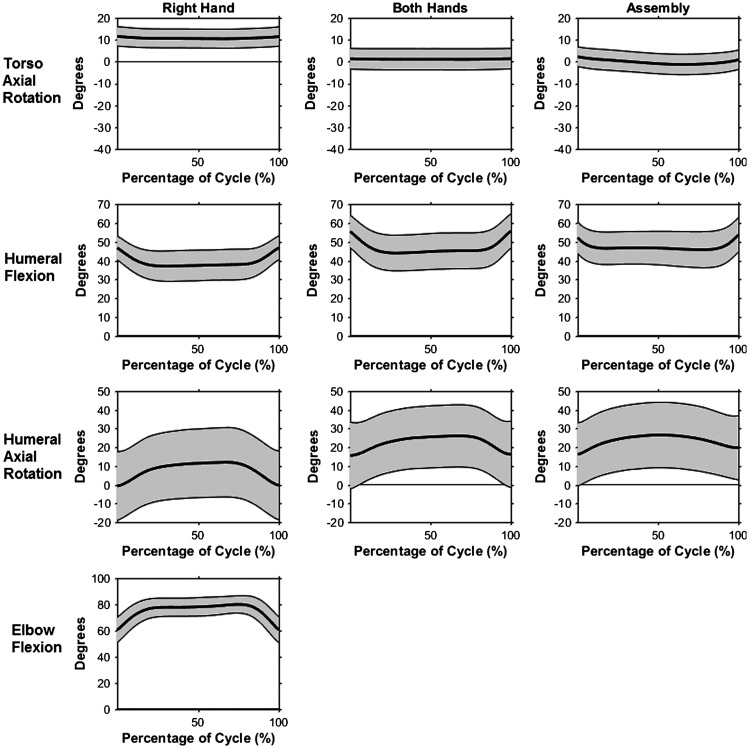
Kinematic profiles with mean (bold line) +/− one standard deviation (gray shaded area) during the right hand, both hands, and assembly FD subtasks: torso + left/−right axial rotation (top), right humeral flexion (second), right humeral + internal/− external axial rotation (third), and right elbow flexion (bottom).

Notes: Flexion decreases slightly during pin placement, while internal rotation increases during pin placement for all subtasks. The pattern of motion for the elbow was the same for all subtasks.

The FD task required minimal movement. Torso flexion angle during the FD subtasks remained at an average of 15.5°, while lateral flexion and axial rotation varied slightly with subtask (Figure 3). Thoracohumeral range of motion within a cycle for all angles was 15° or less. Mean thoracohumeral abduction for both arms decreased from 41.5° in the single hand subtasks to 26.1° during the assembly subtask. Conversely, both mean humeral flexion and mean internal rotation increased from the single hand tasks to the bilateral tasks, from 38.6° and 9.9° to 45.7° and 25.7°, respectively (Figure 3). Average elbow flexion angle was 69.5 and 78.1 for the right and left arms. (Figure 3). Both wrists remained in a nearly neutral posture, with the exception of the left wrist ulnar deviation angle, which was a mean of 15.6° for all subtasks.

### 3.3. Hand and forearm dexterity

**Figure 4.**
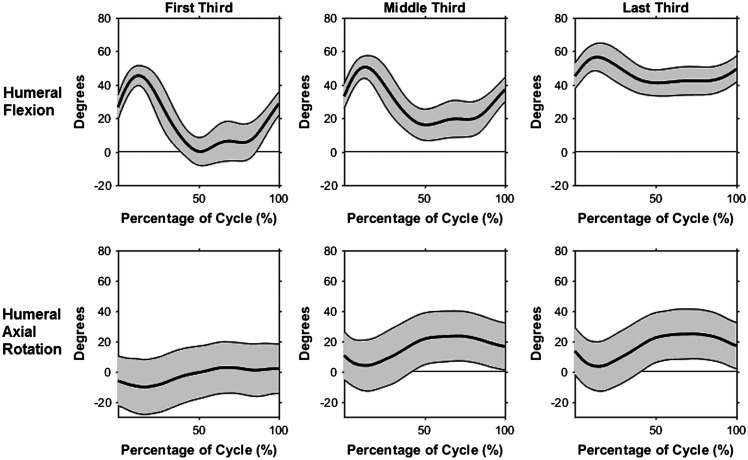
Mean humeral flexion angle and mean humeral axial rotation angle of the right arm with +/− one standard deviation during the HFD placing task.

Note: Both mean flexion and mean internal rotation increase from the first third to the last third.

The first HFD task was the placing task. Torso flexion and lateral flexion remained constant for each cycle at an average of 16.4° of flexion and 3.4° of left lateral flexion. Conversely, average left axial rotation increased from the first third (2.96°) to the last third (16.89°). The maximum values of the thoracohumeral abduction curve decreased by 12.4° from the first third to the last third (Figure 4). Mean flexion angle increased from 17.7° to 46.2° and mean axial rotation changed from 2.6° of external rotation in the first third, to 15.8° and 16.7° of internal rotation in the middle and last third, respectively (Figure 4). Elbow and wrist angles did not change across the placing task trials. The minimum angle of the mean elbow flexion curve was 45.7°, while maximum flexion angle was 106.1°. Both wrist flexion/extension angle and ulnar/radial deviation remained close to neutral for the entire placing task.

**Figure 5.**
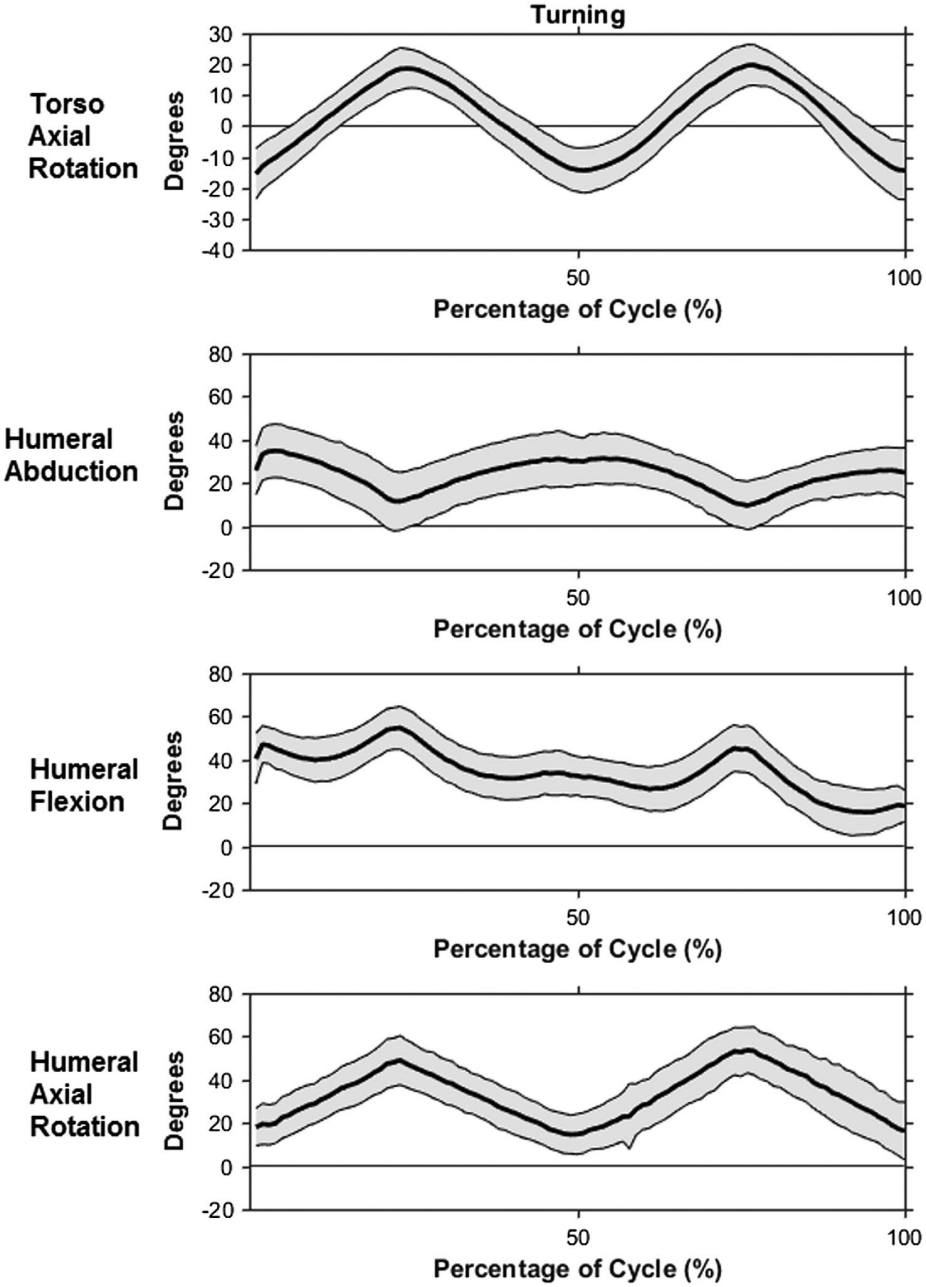
Kinematic profiles with mean (bold line) +/− one standard deviation (gray shaded area) during hand and forearm turning subtask: torso + left/− right axial rotation (top), right humeral abduction (second), right humeral flexion (third), right humeral + internal/− external axial rotation (bottom).

Note: Peaks in torso and humeral axial rotation occur when the hands are at the edges of the board.

The second task in the HFD task was the turning task. Torso flexion remained around 19.8°, lateral flexion was approximately neutral and axial rotation ranged from 22.1° of left rotation to 19.3° of right rotation (Figure 5). The thoracohumeral abduction curve ranged from of 1.82° to 38.06° and mean flexion angle decreased from 40.5° at the beginning of the task to 18.6° at the end, while humeral internal rotation varied between 20° and 45° (Figure 5). Average elbow flexion angle increased from start to finish, from 75° at the start to 100° at the finish. Both wrists had an average extension angle of 9.8°. Mean ulnar deviation angles were 5.9° and 26.4° for the right and left wrists, respectively.

## 4. Discussion

The establishment of a normative kinematic data-set for upper limb focused FCE tasks is intended to improve return to work decision-making. This comprehensive data-set of upper limb kinematics during reaching and dexterity tasks can provide a comparison data-set for future analyses of kinematics during the performance of these tasks by pathological populations, with use during FCE assessments as only one potential application. The high resolution comparison of movement differences will provide information to evaluators that is intended to enable confident discrimination and identification of potential movement compensations or aberrations. Used in conjunction with normative capacity data, these descriptions of movement provide enhanced clarity regarding the patient’s ability to return to work and any limitations they may have. They expand upon and formalize the current heuristic and difficult to implement guidelines.

### 4.1. Sex

As all the tasks in the current study were scaled proportionally to each participant’s anthropometry, sex differences may be irrelevant. This is consistent with previous work that also investigated sex and stature differences on upper arm tasks (Chaffin et al. 2000; Won et al. 2009).

### 4.2. Kinematics

The tasks in this study were specifically chosen to simulate work tasks and to test motion relevant to upper limb function, however the kinematic requirements and demands of these tasks lacked prior evaluation. By classifying the postures using common observations tools such as NIOSH Observation-Based Posture Assessment (NIOSH 2014) and the Rapid Upper Limb Assessment (RULA) (McAtamney & Corlett 1993), a representation of the task demands can help contextualize the motions and postures used by a healthy population.

#### 4.2.1. Torso angles

Torso flexion angle was similar within all reaching and dexterity tasks. Mean torso flexion for all tasks belongs in the NIOSH first category and would be scored up to a three using RULA. These scores are relatively low, indicating minimal required torso flexion.

Although lateral flexion of the torso can result in a negative increase in spine loading (Pope et al. 2002), lateral flexion was not markedly different from neutral for any tasks in this evaluation and remained in the first NIOSH category for all tasks.

Axial rotation was non-neutral for many tasks but would still often be classified in the smallest NIOSH category (0°–30°), with the RR task excepted. Torso axial rotation motion affects injury risk (Marras et al. 1993) and when combined with torso flexion, can increase strain on the spine (Shirazi-Adl et al. 1986). Accordingly, although the current level of axial rotation in most tasks is low, this angle should be closely monitored for potential negative compensations. Also, axial rotation in the RR task is higher than other tasks, indicating that this task would be useful in identifying arm motion deficiencies.

#### 4.2.2. Thoracohumeral angles

Substantial arm elevation was required in all evaluated FCE tasks. Humeral abduction was in the second NIOSH category while humeral flexion was up to the third of five NIOHS categories. In addition, based on the RULA scoring scheme, all tasks would receive up to a four out of seven for greater than 45° elevation while abducted.

Arm elevation is a high risk motion and increasing elevation angle correlates with increased incidence of shoulders injuries (Svendsen 2004; Silverstein et al. 2008). Nevertheless, many different occupations require high levels of arm elevation (Punnett et al. 2000; Frings-Dresen & Sluiter 2003; Svendsen 2005). Therefore, shoulder abduction and flexion are important motions and postures to assess. These tasks allow evaluators to test abilities and movement strategies in these planes. Humeral abduction and flexion outside of the normative profiles, for instance a decreased ability to elevate the arm to reach the equipment, would indicate potential compensations and contraindications to return to work.

Humeral axial rotation is also an important movement to analyze when assessing shoulder motion, although it is difficult to observe and is not included in RULA scoring. In FD and HFD tasks, internal rotation increased during pin and block placement, observable as a lift of the elbow, to the second category (30°–60°). Patients with subacromial disorders would likely avoid this posture because the increasing internal rotation in conjunction with the abducted humeral posture places the arm in a position that is associated with impingement (Brossmann et al. 1996). Those with impingement performing this FCE protocol could use a more externally rotated humerus or increase motion at joints other than the shoulder to avoid this impingement position, subsequently increasing load on those structures (Lomond & Côté 2011).

The motion and postures used by a healthy population in these FCE tasks supports use of these tasks in evaluating upper limb abilities. Shoulder-injured individuals would likely exhibit compensations because of the high demands on the shoulder in these tasks. Some of these, such as avoiding placing the arm in positions that would cause or exacerbate impingement (Brossmann et al. 1996; Kessel & Watson 1997) were discussed but other mechanisms are possible. For instance, in the RR, a large range of both humeral abduction and flexion occur in a short time period, averaging 60 reaches in a minute. This range of motion may be unavailable to shoulder injured individuals (McClure et al. 2006) and thus, they could compensate by increasing torso axial rotation in order to still reach to the same relative position or decreasing the speed of reaching and subsequent segment velocities (Coté et al. 2005).

#### 4.2.3. Elbow angles

According to the observation tools, most of the elbow motion during the reaching and dexterity tasks is relatively low risk. Elbow flexion would be scored only a one on the RULA during the FD task, while the RR and HFD tasks would receive a two. However, using the NIOSH categories, all reaching and dexterity tasks classified into the fourth categories (90°–120°). The NIOSH categories would suggest that as the elbow moves from neutral, the posture becomes higher risk, seemingly in conflict with the RULA guidelines. Grandjean and Kroemer (1997) noted that it is best practice for both strength and skill for the elbow to be bent at right angles, so a bent elbow posture is considered the preferred posture for the elbow in this investigation.

Elbow angles throughout most tasks were within the strongest, most comfortable region. This suggests these tasks are ergonomically sound when considering the elbow. Because these tasks are not elbow demanding, they would not be as effective for screening elbow injuries. Instead, time series elbow joint angles are likely more useful for identifying compensations for adjacent joints.

#### 4.2.4. Wrist angles

The tasks studied required relatively high demand postures from the wrist. Wrist flexion/extension angle often scored up to a three from RULA and received a ‘non-neutral’ classification from the strain index, a commonly used lower arm evaluation tool (Moore & Garg 1995). Similarly, almost every task required some level of wrist ulnar deviation. The ulnar deviation angles recorded in this study would likely be often classified as ‘non-neutral’ or ‘marked deviation’ on the strain index (Moore & Garg 1995) for all tasks due to the low maximum range of motion of the wrist. Ulnar deviation has been implicated in the development of carpal tunnel syndrome and other cumulative trauma disorders of the wrist (Tanaka et al. 1995; Oatis 2004); as such this level of ulnar deviation in nearly all tasks indicates that this angle should be carefully watched by evaluators for any abnormalities.

Wrist postures in these tasks are concerning, as the healthy participants often deviated from neutral. Depending on the patient and the job that they are returning to, these postures could lead to injury (De Krom et al. 1990). It is unclear how cueing patients to keep a neutral wrist would change kinematics at the rest of the joints but the wrists should be monitored during tasks for even greater deviation.

### 4.3. Application of normative profiles

The normative profiles developed provide high-resolution characterization of upper limb motion during generic reaching and dexterity tasks. These can also be used as guides in an FCE setting. With the new description of normative kinematics, comparison to kinematics from known pathological groups is possible. Identified divergences in these datasets can help to create more robust, higher resolution guidelines for observation of motion during FCEs.

**Figure 6.**
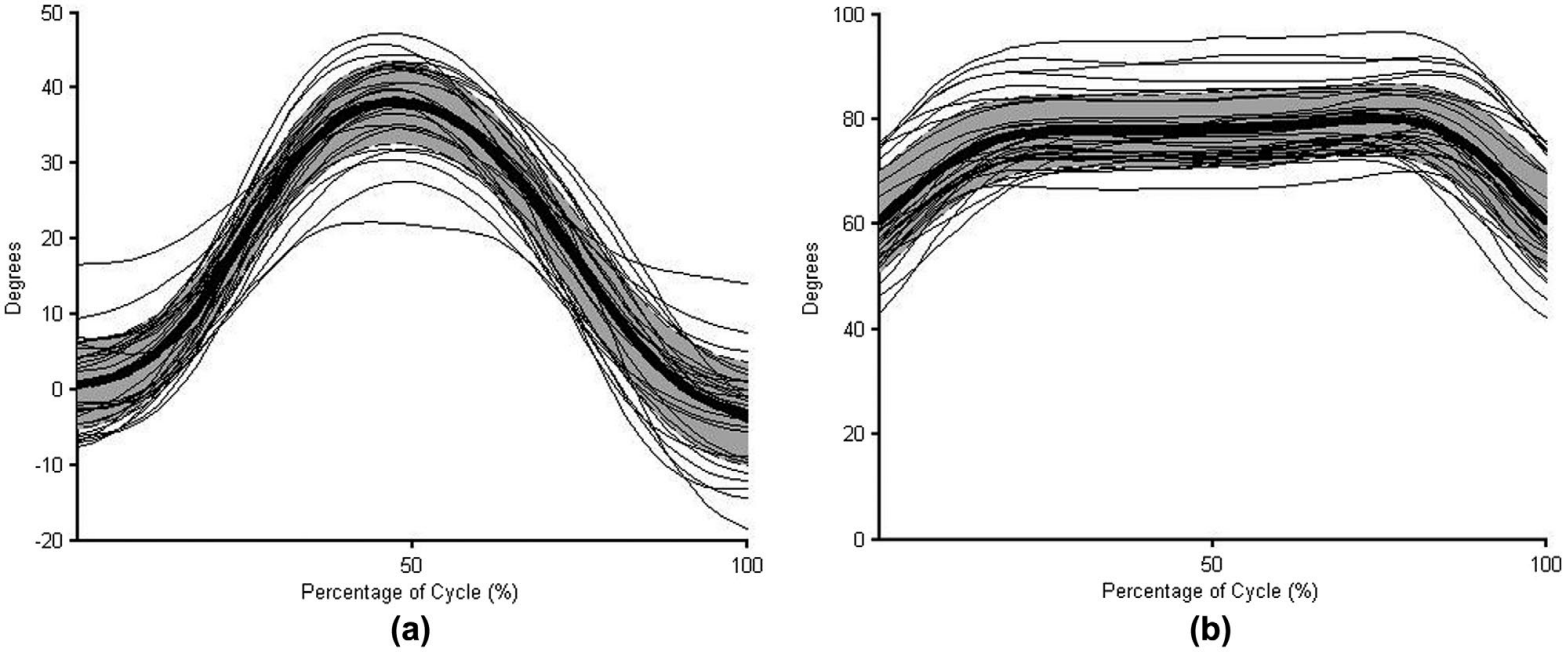
Individual raw mean angle curves overlaying the normative profiles of torso axial rotation in the RR task (a) and elbow flexion during the FD (b) tasks with the mean of the profiles in bold and +/− one standard deviation shaded in gray.

The normative profiles developed in this investigation represent the mean and +/− one standard deviation for a young, healthy population. Thus, approximately 68% of the young (under 25), healthy population could use movement strategies that fall within the normative profiles, meaning that some healthy individuals could use motion outside the standard deviation bands of the profiles. However, the curves of those individuals that differ from the group profiles would likely have the same trend and shape as the representative profiles (Winter 2009). To demonstrate this, each participant’s raw mean curve is plotted against the mean and standard deviation profile for different joints and tasks (Figure 6). For all examples, the shape of the curves and trend of the movement is consistent for all participants, even if the raw magnitudes vary. Further research is needed to determine if these curves, both magnitude and shape, are representative of other age groups.

**Figure 7.**
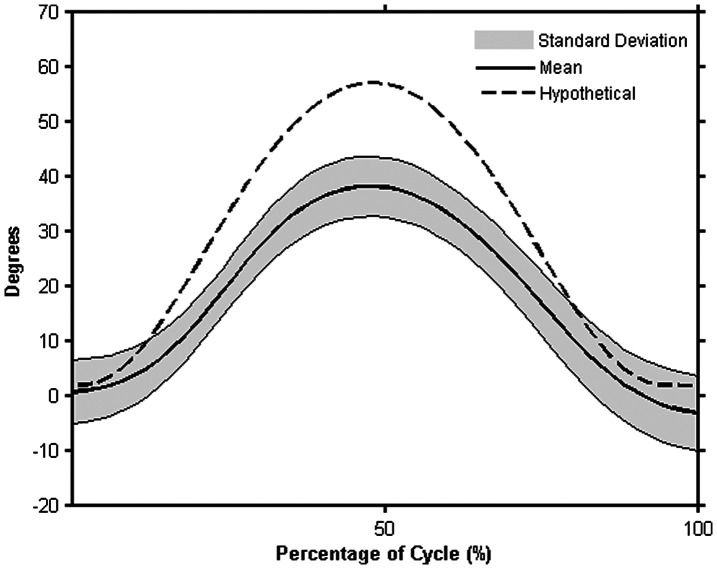
Hypothetical comparison of pathological torso axial rotation (dotted line) to the normative axial rotation profile generated in the current study during the RR task with the mean of the profiles in bold and +/− one standard deviation shaded in gray.

It is likely injured patients profiles would not match the normative profiles (Winter et al. 1990). Creating kinematic profiles from known injury populations or different age groups would allow for a more quantitative comparison of the curves to the healthy profiles. For instance, using the previous RR task example, if a patient had a shoulder injury that decreased their available range of motion (McClure et al. 2006), it is possible that torso axial rotation would increase to compensate for the lack of shoulder motion (Lomond & Côté 2011). To illustrate this, a hypothetical example of the torso axial rotation of the injured patient is contrasted to the normative profile (Figure 7). It is also possible that a person with a known injury could have movement profiles that fall within the normative curve. If their capacity scores also indicate that they are performing at a level that would allow them to return to work, this information together could suggest that the patient is ready to return to work in a decreased capacity (i.e. ‘light duty’).

Individuals could exhibit motion outside of the normative profiles with or without impairment. The implications of the deviations are dependent on the patient and their potential injury. Observation or measurement of deviations from normal would direct evaluators to review the known injury or impairment of the patient being evaluated and to observe motion at other joints in the kinematic chain in order to better understand the implications of deviation. If the trend or shape of motion is consistent with the normative profiles but raw magnitudes differ, it is possible that the patient may be part of the 32% of the healthy population not represented in the normative profiles. On the contrary, if deviations in trend or trajectory of motion are noted, these could be an indication of injury and impairment that could contraindicate return to work.

The goal of FCE’s are to return patients to work as soon as it is safe, because increased time off work is associated with negative effects on both mental and physical health (Strong et al. 2002). Improving the objective determination of safe return to work is a constant pursuit of FCE assessments, particularly in workers’ compensation cases (Hazard et al. 1992; Marmer et al. 2002; Reneman, Fokkens, et al. 2005). Therefore, the addition of objective confirmation based on kinematic data can increase not only the confidence in an evaluator’s decision that a patient is or is not ready to start the return to work process but can also increase the confidence of the patient of their ability to return to work without re-injury.

### 4.4. Limitations

This study had some limitations. Primarily, the FCE tasks were performed in a laboratory setting, instead of a clinical one, with task instructions given by a graduate student and not a trained FCE administrator. Clinical procedures were followed as closely as possible, but performance could have been affected by the environment (Matheson et al. 1992; Fasoli et al. 2002). It should also be noted that while the purpose of this study was to create normative kinematic profiles from a young, healthy population, the majority of the participants in this study were students or had office jobs. A population of the same age but in a different occupational category could have different strategies to complete these tasks due to task familiarity (Faber et al. 2011). Finally, there is a high degree of variability in the kinematic outcomes reported; this is a likely a result of the nature of the FCE tasks. These are function-oriented tasks chosen to represent work activities and, as such, have an inherent degree of variability that is captured in these data, allowing for improved understanding and interpretation of upper limb motion during performance of these particular tasks.

## 5. Conclusions

• The primary contribution of this investigation was to quantitatively examine and characterize the kinematics of a young, healthy population during upper extremity focused Function Capacity Evaluation (FCE) tasks. These data provide novel practical guidance for understanding and identifying healthy or normal movement during these tasks.
• The most important outcome of this investigation is the comprehensive data-set of upper limb and torso kinematics in these FCE tasks. Because these tasks are simulations of work tasks intended to evaluate motions relevant to common work tasks, these data are crucial for improving movement assessment of related tasks in both clinical and ergonomic settings.
• The kinematics of these tasks indicate their utility as evaluation tools for assessing the upper limb, and specifically the shoulder and wrist, as the highest demand postures and largest range of motion are required in these areas. It is likely that injured patients will exhibit kinematics or movement strategies different from the healthy control group that can be more consistently identified through comparison to the normative data.

## Disclosure statement

No potential conflict of interest was reported by the authors.
